# A Potential Biocontrol Agent *Streptomyces* *violaceusniger* AC12AB for Managing Potato Common Scab

**DOI:** 10.3389/fmicb.2019.00202

**Published:** 2019-02-08

**Authors:** Arslan Sarwar, Zakia Latif, Songya Zhang, Jianjun Hao, Andreas Bechthold

**Affiliations:** ^1^Department of Microbiology, Faculty of Life Sciences, University of Central Punjab, Lahore, Pakistan; ^2^Department of Microbiology and Molecular Genetics, University of the Punjab, Lahore, Pakistan; ^3^Department of Pharmaceutical Biology and Biotechnology, Institute of Pharmaceutical Sciences, University of Freiburg, Freiburg im Breisgau, Germany; ^4^School of Food and Agriculture, The University of Maine, Orono, ME, United States

**Keywords:** *Streptomyces scabies*, biological control, plant growth promoting *Streptomyces*, antagonistic *Streptomyces*, potato common scab

## Abstract

Potato common scab (PCS) is an economically important disease worldwide. In this study we demonstrated the possible role of *Streptomyces violaceusniger* AC12AB in controlling PCS. Isolates of *Streptomyces scabies* were obtained from CS infected tubers collected from Maine United States, which were confirmed by morphological and molecular analysis including 16S rRNA sequencing and RFLP analysis of amplified 16S-23S ITS. Pathogenicity assays related genes including *txtAB*, *nec1*, and *tomA* were also identified in all *S. scabies* strains through PCR reaction. An antagonistic bacterial strain was isolated from soil in Punjab and identified as *S. violaceusniger* AC12AB based on 16S rRNA sequencing analysis. Methanolic extract of *S. violaceusniger* AC12AB contained azalomycin RS-22A which was confirmed by ^1^H and ^13^C-NMR, ^1^H/^1^H-COSY, HMBC and HMQC techniques. *S. violaceusniger* AC12AB exhibited plant growth promotion attributes including Indole-3-acetic acid production with 17 μgmL^-1^ titers, siderophores production, nitrogen fixation and phosphates solubilization potential. When tubers were inoculated with *S. violaceusniger* AC12AB, significant (*P* < 0.05) PCS disease reduction up to 90% was observed in greenhouse and field trials, respectively. Likewise, *S. violaceusniger* AC12AB significantly (*P* < 0.05) increased potato crop up to 26.8% in field trial. Therefore, plant growth promoting *S. violaceusniger* AC12AB could provide a dual benefit by decreasing PCS disease severity and increasing potato yield as an effective and inexpensive alternative strategy to manage this disease.

## Introduction

Potato common scab (PCS) is considered among top five diseases by potato farmers in United States (Slack, 1991). PCS is caused by Gram positive, filamentous bacteria in the genus *Streptomyces*. *Streptomyces* are soilborne saprophytic bacteria, mostly famous for the production of antibiotics (Kemung et al., 2018). However, only few of them are plant pathogens. Although, several species of *Streptomyces* can cause common scab (CS), *Streptomyces scabies* was considered as predominant plant pathogen (Lambert and Loria, 1989). PCS infection is characterized by superficial, raised or pitted scab lesions on the surface of the tubers. The occurrence of PCS infection is, generally not hazardous to human health. However, they may deteriorate the quality of tubers and effects the market value. For instance, potato industry in Canada reported to experience $1.2 million loss every year due to this disease (Al-Mughrabi et al., 2016).

Scab lesions on the surface of the tubers develop due to a phytotoxin called thaxtomin. In 1898, thaxtomin was firstly described (King et al., 1989; Lawrence et al., 1990) as toxin responsible to produce CS on immature tubers. All PCS causing *Streptomyces* spp. produce thaxtomin A or another member of thaxtomin family (Loria et al., 2008). Biosynthesis of this phytotoxin encompasses non-ribosomal peptide synthetases encoded by *txtA* and *txtB* genes (Loria et al., 2008). The genes responsible for pathogenicity like *txtAB*, *nec1* and *tomA* are clustered together and termed as pathogenicity island (PAI) (Kers et al., 2005). PAI consists of thaxtomin genes including *txtAB* (Healy et al., 2000), *txtH*, *txtC* (Healy et al., 2002), *txtR*, *txtE* (Joshi et al., 2007), *nos/txtD* (Kers et al., 2004), and genes for pathogenicity factors like Tomatinase (*tomA*) and Necrotic protein (*nec1*) (Kers et al., 2005; Barry et al., 2012). Secreted Nec1 protein helps to enhance the virulence by weakening the plant defense mechanism (Bukhalid et al., 1998). *TomA* gene encodes for a virulent protein having high similarity with phytopathogenic fungi tomatinase (Kers et al., 2005).

Although thaxtomin A, which is encoded by *txtAB* gene, is considered as a major player toward plant pathogenicity, other genes including *nec1* and *tomA* are also somehow required for the virulence (Loria et al., 2006). Many unknown factors can play a supportive role in pathogenicity. However, the prevalence of CS pathogens without *txtAB* genes are either very rare or confined to geographic locations (Park et al., 2003; Wanner, 2004).

Over the decades, PCS management remained a serious problem among potato growers. The control strategies are challenging due to limited understanding of genetic diversity of *S. scabies* and genetic differences in various potato cultivars (Dees and Wanner, 2012). Several physiochemical approaches like reducing soil pH, crop rotation, and soil fumigation agents like chloropicrin (trichloronitromethane) have conventionally been used with harmful effects to the environment (Larkin et al., 2011; Xue et al., 2018). In contrast, research in biological control as an alternative strategy is emerging. Several antagonistic bacteria including *Bacillus* spp. (Meng et al., 2013), *Pseudomonas* spp. (Arseneault et al., 2015) and *Streptomyces* spp. (Sarwar et al., 2018) have been used as biocontrol agent against PCS.

The present study was designed to evaluate the pathogens responsible for PCS incidences and to assess the antagonistic ability of *S. violaceusniger*. It was hypothesized that *S. violaceusniger* AC12AB could be used as effective biological control agent due to its ability to promote plant growth and suppress PCS.

## Materials and Methods

### Sample Collection, Bacterial Isolation, and Identification

Potatoes having visible CS symptoms were collected from Presque Isle, ME, United States. All collected samples were carefully transferred to the laboratory at the University of Maine, United States. Samples were stored at 4°C prior to use.

Tubers with CS symptoms were washed and surface sterilized with 5% sodium hypochlorite (NaOCl) for 1 min. Surface sterilized tubers were rinsed with sterile distilled water and air dried. The infected portion from CS tubers were carefully excised with sterile scalpel and triturated to form a homogenized paste by adding 1 mL Tris-HCl. The homogenized suspension was poured into 2 mL Eppendorf tubes, separately. The Eppendorf tubers were placed at 55°C for 2 h to remove unwanted microorganisms. This suspension was ten-fold diluted with sterile distilled water before pouring onto yeast malt extract (YME) agar plates (Shirling and Gottlieb, 1966). An aliquot of 100 μL from diluted suspension was used to spread on YME agar plates and placed in an incubator for 5–7 days at 28°C. After incubation, YME agar plates were checked for the white cottony *Streptomyces* like colonies.

Antagonistic bacterial isolates were collected from agriculture field located at Lahore, Pakistan having no visible CS symptoms over the period of past 5 years. Suppressive soil samples were used to isolate Actinomycetes by serial dilution method (Wang et al., 2015). Colonies were further purified on YME agar plates (Shirling and Gottlieb, 1966). Microorganisms particularly antibiotic producing actinomycetes were targeted as promising candidate as PCS antagonistic bacteria (Kharel et al., 2010).

### DNA Extraction and PCR Amplification

Selected bacterial spores were inoculated into YME broth and incubated for 3 days in shaking incubator with 180 rpm at 28°C temperature. After incubation, supernatant was separated from cell pellet by centrifugation. The cell pellet was used for genomic DNA extraction by using the FastDNA^®^ kit (MP Biomedicals, Santa Ana, CA, United States). PCR amplification of DNA samples were performed with 16S rRNA primers (Edwards et al., 1989). PCR reaction was performed in PCR tubes with 25 μL reaction volume which included 1 μL (50ng) DNA (A_260_/A_280_ ratio was 1.9) template, 5 μL 5X PCR buffer, 0.50 μL 10 mM dNTPs, 0.50 μL 10 μM forward and reverse primers each, 0.10 5 u/μL Taq polymerase and 17.90 μL H_2_O. PCR reaction was programmed as, initial denaturation for 5 min at 95°C followed by 30 cycles of denaturation for 30 s at 95°C, annealing for 40 s at 60°C, extension for 40 s at 72°C and final extension was performed for 5 min at 72°C.

### Identification of PCS Pathogens

*Streptomyces* species specific primers were used for the identification of PCS causing pathogens. Species specific primers for *Streptomyces* pathogens including *S. scabies* (Lehtonen et al., 2004), *S. europaeiscabiei*, *S. bottropensis*, *S. stelliscabiei* (Wanner, 2006), *S. acidiscabiei*, and *S. turgidiscabiei* (Tagawa et al., 2008) were used for the identification by PCR amplification. Amplification of 16S-23S internal transcribed spacer (ITS) sequence was performed with ITS forward and reverse primers (Song et al., 2004). PCR amplified product was digested with *Hpy99I* restriction enzyme which expurgated the amplicon at 1629–1633 nucleotide position.

DNA fragments were visualized under gel electrophoresis. PCR amplified product was sent to DNA sequencing facility, University of Maine for sequencing. 16S rRNA sequences were submitted to NCBI to obtain accession numbers.

Polymerase chain reaction was used to amplify PAI related genes including *txtAB*, *nec1*, and *tomA* (Bukhalid et al., 1998; Wanner, 2006). PCR reaction conditions were same as above except the annealing temperature was adjusted at 60°C, 55°C and 48°C for *nec1*, *tomA*, and *txtAB* genes, respectively.

### Disk Diffusion Assay

Antagonistic *Streptomyces* spp. were checked against PCS pathogens by disk diffusion assay (Clinical and Laboratory Standards Institute, 2015). Pure cultures of twelve antagonistic *Streptomyces* spp. were prepared by inoculating few spores into 100 mL YME broth and incubated at 28°C in shaking incubator at 180 rpm for 5–7 days. The broth culture was centrifuged at 9,000 ×*g* and supernatant were used for preparation of methanol extract. Meanwhile, YME broth cultures of PCS pathogenic *Streptomyces* strains were spread on YME agar plates with Rattler^TM^ plating beads (Zymo Research Cooperation, United States), separately. 25 μL methanolic extract of antagonistic *Streptomyces* were poured on filter paper disks and placed on YME- agar plates previously spread with pathogenic PCS suspension. The plates were incubated for 48–72 h at 28°C. After incubation, clear zone around filter paper disks were checked and results were recorded in mm.

### Plant Growth Promotion

Twelve antagonistic *Streptomyces* spp. were evaluated for plant growth attributes including indole-3-acetic acid (IAA) production, phosphate solubilization, siderophores production and *in vitro* nitrogen fixation. IAA production was estimated by colorimetric method (Gordon and Weber, 1951; Amin and Latif, 2017) and confirmed by HPLC-DAD-MS as mentioned by Sarwar et al. (2018). The IAA production titer from antagonistic *Streptomyces* spp. was performed by observing optical density (O.D_530nm_) against standard curve of IAA and recorded in μg mL^-1^ (Bric et al., 1991). Phosphate solubilization was assessed by the method previously described by Sylvester-Bradley et al. (1982). Glucose yeast medium (GY) along with two solutions; one containing 10% 50 mL K_2_HPO_4_ and second solution containing 10% 100 mL CaCl_2_ were prepared and added in 1 L GY medium (Ambrosini et al., 2012). The medium was autoclaved and poured into petri plates after cooling. The addition of two solutions made an opaque insoluble layer of CaCl_2_. The plates were inoculated with antagonistic *Streptomyces* isolates, separately and incubated for 7 days at 28°C. After incubation, inhibition zone was observed and recorded.

Siderophores production was checked by inoculating bacterial spores on chrome azurol S (CAS) agar plates as mentioned by Schwyn and Neilands (1987). After the incubation of 5–7 days at 28°C, development of yellow to orange color was observed.

Nitrogen fixation potential of antagonistic *Streptomyces* spp. was examined by acetylene reduction assay (ARA) as described by Rice and Paul (1971). Nitrogen free mannitol (NFM) medium (Doty et al., 2009) slants were prepared in glass tubes and inoculated with antagonistic *Streptomyces* spp., separately. The tubes were sealed with a stopper and head space was filled with 2% oxygen. About 10% head space was exchanged with equal amount of acetylene. The tubes were placed in an incubator at 28°C for 2 weeks. Reduction of acetylene to ethylene was measured by gas chromatography (Agilent technologies 7890A GC system), which was equipped with flame ionizing detector and Agilent CP7348 column (25m × 0.25mm). As a positive control, two bacterial strains belonged *Bacillus amyloliquefaciens* (ZM2; accession number JX185642) and *Pseudomonas aerouginosa* (ZS24; accession number JQ990311) were used (kindly provided as positive control by Dr. ZL, University of the Punjab, Pakistan).

### Extraction and Analysis of Bioactive Compounds by HPLC-DAD-MS

Antagonistic *Streptomyces* strains were inoculated in 150 mL YME broth in a 500 mL shaking flask. The flasks were incubated for 3 days at 28°C in an incubator shaker with 180 rpm. After incubation, the culture was centrifuged, pellet was discarded, and supernatant was undergone twice extraction with equal amount of ethyl-acetate. The extract was concentrated *in-vacuo* and re-suspended in methanol. For the HPLC-DAD-MS analysis, Agilent 1100 system was used equipped with a XBridge C-18 (3.5 mm, 100 mm × 4.6 mm) reverse phase column, a diode array detector and a quadrupole mass detector. An aliquot of 20 μL diluted crude extract was injected into the HPLC system and eluted isocratically with 95:5 methanol/water at a 0.5 mL min^-1^ flow rate.

### Purification and Structural Elucidation of Azalomycin

Culture of *S. violaceusniger* strain AC12AB (100 mL) was used to inoculate in 10 L YME broth at 28°C for 5 days in an incubator shaker with 150 rpm. After incubation, the culture was sonicated for half an hour. The culture was then centrifuged at 11,200 ×*g*, supernatant was used for extraction with equal amount of ethyl acetate. The ethyl acetate extract was concentrated *in-vacuo* and powdered extract was re-suspended in methanol. The methanolic extract was used for thin layer chromatography (TLC) and silica gel column chromatography with 5:1 dichloromethane and methanol buffer system. All the fractions were analyzed for their biological activity against *S. scabies* and most active fraction was further purified by Sephadex LH-20 column chromatography system with methanol as mobile phase. The fraction was analyzed by HPLC-DAD-MS system and further purification was performed by SPE Oasis^®^ HLB20 35 cc cartridge (6 g). Fractions were eluted in SPE column with step gradient (20–100%) of methanol. Purified fraction was obtained after final purification with semi-preparative HPLC.

Final purification was achieved with help of semi-preparative HPLC system (Agilent 1100 Series). In HPLC system, as a stationary phase Zorbax B-C C18 (9.4 mm × 20 mm) main column and Zorbax B-C18 (9.4 mm × 150 mm) pre-column was used. The compound was eluted with buffer A (acetonitrile/acetic acid 0.5%) and buffer B (water/acetic acid 0.5%) with 2 mL min^-1^. Methanolic extract purified from SPE Oasis^®^ HLB20 35 cc cartridge was spiked on the column. 6mg purified azalomycin obtained from semi-preparative HPLC system, was dissolved in CD_3_OD and analyzed for one dimensional NMR including ^1^H (400 MHZ) and ^13^C-NMR (100 MHZ) and 2-D NMR including HMQC, ^1^H/^1^H-COSY and HMBC on a Bruker DRX-500 NMR spectroscopy (Bruker, Karlsruhe, Germany).

### Plant Growth Promotion and Pathogenicity Assay on Potato Tubers

#### Greenhouse Assay

Pathogenicity assay on tubers was performed in greenhouse assay (Wanner, 2006). From eighteen isolates of pathogenic *Streptomyces* spp., two isolates namely *S. scabies* strain AJ-7 (Accession number MG725948.1) and AJ-10 were selected whereas *S.* *violaceusniger* strain AC12AB (Accession number MH388022.1) was used as antagonistic and plant growth promoter strain. As a positive control PCS pathogenic *S. scabies* strain AC-46 (Accession number KU560917.1) was also used. To observe normal growth pattern, tubers were also inoculated without any bacterial inoculation. The greenhouse assay was performed twice at greenhouse facility, University of Maine, Orono, United States during 2016-2017. Pathogenic *S. scabies* AJ-7, AJ-10, AC-46, and antagonistic *S. violaceusniger* AC12AB were cultivated separately in YME broth for 3–5 days at 28°C in incubator with shaking at 150 rpm, until they attained 10^6^ CFU mL^-1^ conc. After incubation, cultures were separately centrifuged at 9,000 ×*g*. Supernatant was discarded, and bacterial cell mass was re-suspended in sterile distilled water to prepare inocula with 10^6^ CFU mL^-1^ conc. Pots were filled with compo Sana Universal^®^ (Munster, Germany). Tubers were surface sterilized with 5% NaOCl for 5–10 min and washed with sterilized water. Washed tubers were sown into respective five pots as replicates. After 2–3 weeks of sowing, pots were inoculated by drenching with 100 mL bacterial suspension. The average temperature was maintained between 25 and 28°C. The plants were kept hydrated and continuously monitored for the increase in shoot, root length, tuber weight and decrease in PCS symptoms for 3 months. After harvesting, potato tubers were evaluated against growth and disease parameters. The results were recorded and pathogenic *Streptomyces* spp. was re-isolated from CS infected tubers to confirm the source of CS infection.

#### Field Trial

Field trial was conducted to determine disease suppression and plant growth promotion of antagonistic *S.* *violaceusniger* AC12AB. Field trial was conducted in a field available at University of the Punjab, Lahore Pakistan. Indigenous CS pathogen *S. scabies* AC-46 and antagonistic *S.* *violaceusniger* AC12AB were used. Inoculum of pathogenic and antagonistic strains were prepared as mentioned above. Disease free tubers (cv. Berna; Purchased from Punjab Seed Corporation, Pakistan) were disinfected with 5% NaOCl and washed with sterile water. Tubers were sown in a randomized complete block design in duplicates.

Each block (62 square feet) contained four rows; the length of each row was 1.5 m with 2.5 m distance between each block. Six potato seeds were implanted into each row. After 2–3 weeks of plantation, bacterial spore suspension (prepared as described above) was drenched 20–30 cm deep into the plant’s roots. Plants were watered as required under natural light and temperature. Plants were monitored for growth in shoots and roots length. After harvesting, tubers were evaluated for decrease in CS symptoms, increase in tuber weight and yield acre^-1^ were recorded.

#### Statistical Analysis

All the experiments were performed in triplicates and *P* < 0.05 was considered as statistically significant. The results were subject to one-way analysis of variance (ANOVA) and compared means were separated by Tukey’s test. Statistical analysis was performed by using SPSS software (IBM SPSS Statistics, version 21).

## Results

### Identification and Molecular Characterization of *Streptomyces* Causing PCS

Eighteen bacterial isolates were analyzed by PCR amplification of 16S rRNA gene, out of which, all isolates were confirmed as *Streptomyces*. *Streptomyces* isolates were further identified by PCR amplification with using species specific primers, PCR amplification of 16-23S ITS region with ITS primers and digesting the amplicon with *Hpy99I* enzyme. After RFLP pattern analysis, all eighteen *Streptomyces* isolates were found to belong *S. scabies* (Flores-González et al., 2008).

PCR was also performed to identify the pathogenicity-related genes in *Streptomyces* isolates, which were subject to PCR amplification of *txtAB*, *nec1* and *tomA* genes. All *Streptomyces* isolates were found to contain *txtAB*, *nec1*, and *tomA* genes. Antagonistic bacterial isolates were also screened for *txtAB*, *nec1* and *tomA* genes; but, did not test positive for those genes which showed that antagonistic bacterial isolates do not produce thaxtomin A and are non-pathogenic. Among antagonistic bacterial isolates, one bacterial strain AC12AB was identified as *S. violaceusniger* after 16S rRNA analysis with MH388022.1 accession number.

### Antagonistic Potential and Isolation of Azalomycin From *Streptomyces violaceusniger* AC12AB

**FIGURE 1:**
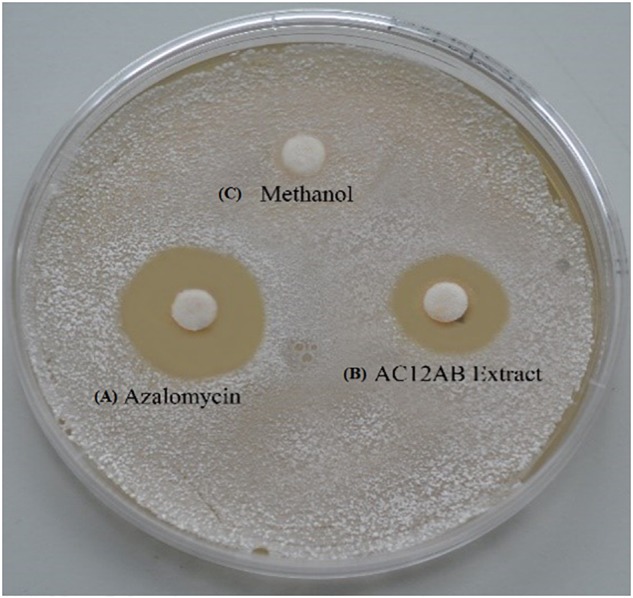
Inhibition of *S. scabies* (AJ-7) by disk diffusion assay. *Streptomyces scabies* (AJ-7) was grown on YME agar plates with filter paper disks containing **(A)** purified azalomycin dissolved in methanol; **(B)** *Streptomyces violaceusniger* AC12AB crude extract; **(C)** methanol only.

Disk diffusion assay was performed to determine the antibacterial potential of antagonistic *Streptomyces* isolates. The bacterial extract from *S. violaceusniger* AC12AB had high inhibitory activity (18 mm) against *S. scabies* (AJ-7) (Figure 1 and Supplementary Table S1).

### Plant Growth Promoting Potential of *Streptomyces violaceusniger* AC12AB

**FIGURE 2:**
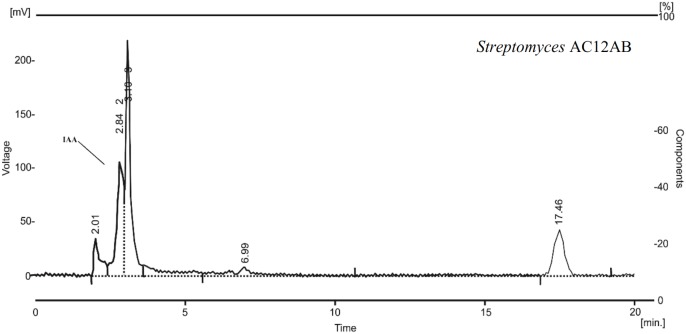
HPLC chromatogram of *S. violaceusniger* AC12AB showing indole-3-acetic acid (IAA) peak at 2.84 min retention time.

*Streptomyces violaceusniger* AC12AB was analyzed to produce plant growth promoting attributes including IAA, phosphate solubilization, siderophores production and *in vitro* nitrogen fixation. With colorimetric method, the highest potential of IAA production was estimated in case of *S. violaceusniger* AC12AB as 17 μgmL^-1^ after 4 days of incubation at 28°C. Production of IAA was also confirmed by HPLC analysis (Figure 2). From twelve antagonistic bacterial strains, three antagonistic *Streptomyces* isolates (*Streptomyces* A1RT, *S. violaceusniger* AC12AB and *Streptomyces* A-1; data not shown except *S. violaceusniger* AC12AB) were positive for siderophores production by producing blue color around bacterial cultures in NFM medium (Supplementary Figure S1). Phosphate solubilization test was performed with antagonistic bacterial strains and only *S. violaceusniger* AC12AB and *Streptomyces* A1RT exhibited clear zone around bacterial colonies (Supplementary Figure S2). ARA was performed against antagonistic bacterial isolates. The maximum value for ARA 4351.0 nMole/24h was recorded from *S. violaceusniger* AC12AB. However, 2278 and 1549 nMole/24h ethylene production were estimated from *Streptomyces* A1RT and *Streptomyces* A-1 strains, respectively (Supplementary Table S2).

### Purification and Structural Elucidation of Bioactive Compound

**FIGURE 3:**
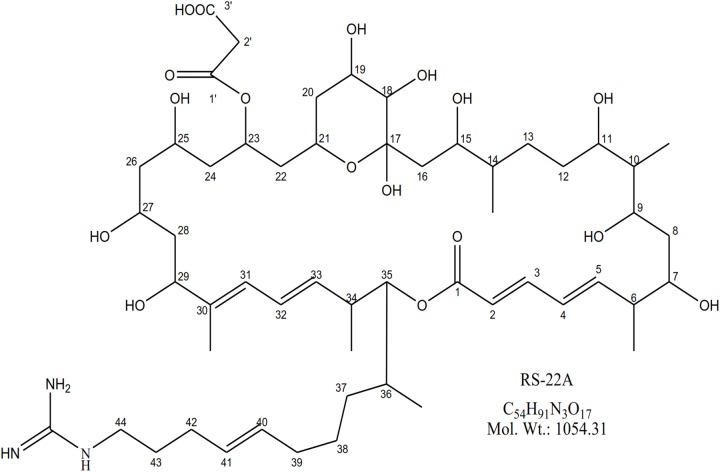
Predicted structure of azalomycin RS-22A purified and isolated from *Streptomyces* AC12AB.

Through a targeted mass fractionation, a white colored amorphous powder was purified having strong antibacterial activity against *S. scabies*. The molecular mass of the compound was predicted to be C_54_H_91_N_3_O_17_ based on observed molecular ion [M-H]^-^; *m/z* 1054.5 (Supplementary Figure S3). Analysis of one-dimensional NMR (^1^H and ^13^C NMR spectra) indicated ten olefinic carbons, twelve oxy-methine and one quaternary hemiacetal carbon (Supplementary Figures S4, S5). Analysis of 2D-NMR revealed the characteristic guanidine carbon (Supplementary Figures S6–S8 and Supplementary Table S3). The absorbance spectrum of compound (Supplementary Figure S9) exhibited distinct maxima 250–300 nm closely related to azalomycin (Figure 3) analog RS-22A (Ubukata et al., 1995).

### Plant Growth Promotion and PCS Disease Suppression Under Greenhouse and Field Conditions

**FIGURE 4:**
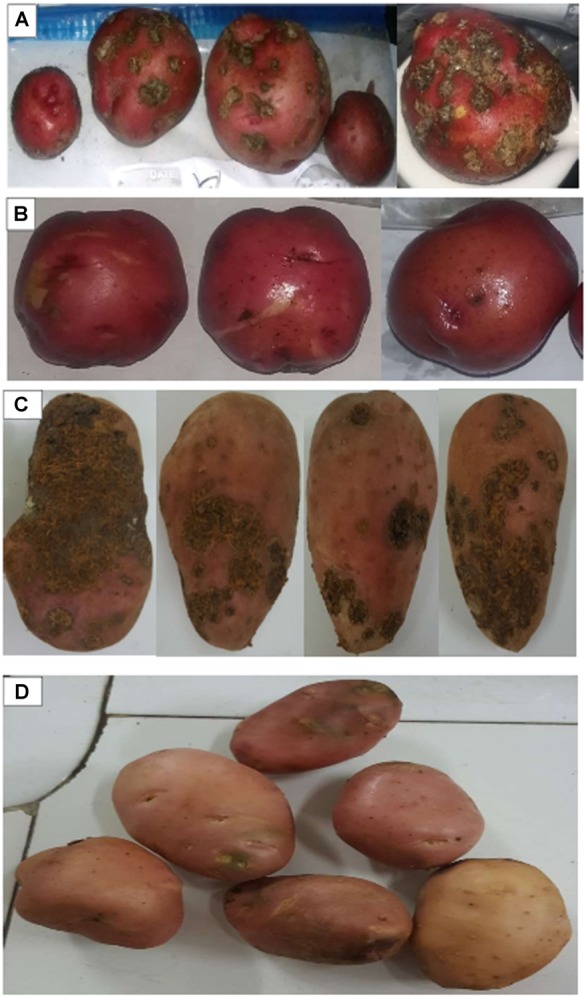
Tubers harvested from greenhouse assay and field trial. **(A)** Tubers harvested from after inoculation with *S. scabies* AJ-7 in greenhouse assay. **(B)** Tubers harvested after inoculation with *S. scabies* AJ-7 + *S. violaceusniger* AC12AB in greenhouse assay. **(C)** Tubers harvested after inoculation with *S. scabies* AC-46 in field trial. **(D)** Tubers harvested after inoculation with *S. scabies* AC-46 + *S. violaceusniger* AC12AB in field trial.

**FIGURE 5:**
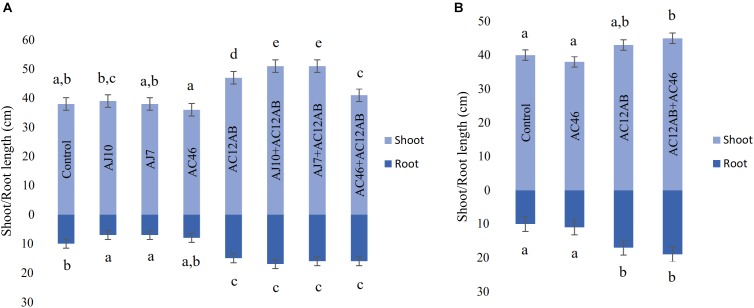
Effect of *S. violaceusniger* AC12AB on potato growth. **(A)** Root and shoot growth in greenhouse trial. **(B)** Root and shoot growth in field trial. Error bars representing ± SE. Measurement was represented by mean ± SE of triplicates. Means followed by different letters show significant differences detected by Tukey’s test at a significance level *P* < 0.05.

**FIGURE 6:**
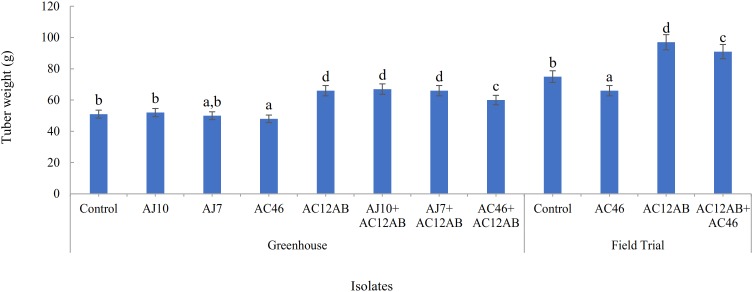
Effect of *S. violaceusniger* AC12AB on potato tuber, which were inoculated with one of *S. scabies* isolates (AJ10, AJ7, and AC46) in greenhouse and field trials. Statistical analysis for greenhouse and field trial was calculated separately. Tuber weight was represented by mean ± SE of triplicates. Means followed by different letters show significant differences detected by Tukey’s test at a significance level *P* < 0.05.

In greenhouse assay, pathogenic *S.* *scabies* isolates (AJ-7, AJ-10, and AC46) caused CS lesions on potato tubers (Figure 4A,B and Table 1). The inoculation with antagonistic *S. violaceusniger* AC12AB significantly reduced DS index (*P* < 0.05) (Table 1). There were 47, 24.6, and 41% increases in shoot length, root length and tuber weight, respectively, when *S. scabies* AJ10 was used in combination with *S. violaceusniger* AC12AB (*P* < 0.05) (Figure 5, 6). Field trial using *S. violaceusniger* AC12AB revealed 83% disease reduction (Figure 4C,D and Table 1), 26.8% yield increase (Table 1), and significant (*P* < 0.05) increase in plant growth attributes (including increase in shoot/root length, number of tubers and tuber weight) (Figure 5, 6).

**Table 1:** Effect of growth promoting *Streptomyces* on potato (*Solanum tuberosum*) grown under greenhouse at the University of Maine, Maine, United States and field conditions in University of the Punjab, Lahore, Pakistan.

Treatment	Greenhouse		Field trial		Field trial	
	DS Index	% decrease	DS index	% decrease	Yield (Kg/h)	% increase
*Streptomyces scabies* (AJ-7)	153 ± 1f	**–**	N/A	N/A	N/A	N/A
*Streptomyces scabies* (AJ-10)	181 ± 1g	–	N/A	N/A	N/A	N/A
*Streptomyces scabies* (AC-46)	96 ± 1e	–	78 ± 0.5 c	–	7,650 ± 1 b	3.37 a
*Streptomyces violaceusniger* (AC12AB)	0.2 ± 0.01a	–	0 ± 0.01 a	–	9,100 ± 1 c	18.8 b
AJ7+AC12AB	12.6 ± 0.01c	91.70 b	N/A	N/A	N/A	N/A
AJ10+AC12AB	7.9 ± 0.01b	91.77 b	N/A	N/A	N/A	N/A
AC46+ AC12AB	17.4 ± 0.1d	90.30 a	13.2 ± 0.4 b	83.07	9,701 ± 1 d	26.8 c
Control	0.3 ± 0.01a	–	1 ± 0.01 a	–	7,400 ± 1 a	–

*The results are presented as mean of three independent experiments ± SE. N/A, not applicable. Means followed by different letters are significantly different detected by Tukey’s test at a significance level *P* < 0.05.*

## Discussion

In this research, *S. violaceusniger* AC12AB was assessed for its efficacy in suppressing CS disease and plant growth promotion in potato crop. The results indicated that although *S. violaceusniger* AC12AB application reduced the PCS disease up to 83%, their efficacy of plant growth promotion in field trial varied as compared to greenhouse assay.

Field soil is a complex environment that contains multiple factors which are difficult to control. CS development is not only dependent upon bacterial inoculum, but also by other physical and biological factors, including soil condition, irrigation strategy, plant variety, and weather conditions (Lazarovits et al., 2007). In the current study, field trial was conducted in the soil, and the average disease severity index was recorded as 1.1 (in the control). Moreover, dry and hot weather conditions of Pakistan may favor the development of CS infections in the tubers. All these factors could affect the disease outcome and may hinder the disease management under natural conditions. Previously, disease management remained dependent upon the use of chemical pesticides (Hvězdová et al., 2018), maintaining high soil moisture level (Powelson and Rowe, 2008), use of resistant cultivars (Dees and Wanner, 2012) and crop rotation (Larkin et al., 2011). Biological control agents have been extensively studied to control plant pathogens and simultaneously reducing environmental pollution and ecological distribution due to the irrational use of pesticides in fumigation.

Eckwall and Schottel (1997) used *Streptomyces diastatochromogenes* PonSSII as biocontrol agent against PCS by demonstrating antibiosis and competition mechanism. Similarly, Han et al. (2005) and Singhai et al. (2011) used *Bacillus* sp. sunhua and *Pseudomonas* spp. to control CS infections, respectively. Moreover, antimicrobial agents from bacterial spp. such as macrolactin A, iturin A, surfactin, bacillaene, fengycin, isatropolone C and difficidin (Schneider et al., 2007; Chowdhury et al., 2015; Lin et al., 2018; Sarwar et al., 2018) have been used against plant pathogens. In this study, we have identified a novel plant growth promoting *S*. *violaceusniger* AC12AB, which was confirmed to be an effective and inexpensive method to control PCS and simultaneously enhance the crop yield. PCS management remains unsolved as there is lack of chemical products. In this study, *S*. *violaceusniger* AC12AB exhibited strong antibacterial activity against *S. scabies*. Further analysis by NMR revealed that the main bioactive compound produced by *S*. *violaceusniger* AC12AB was azalomycin RS-22A, which has been previously used as a broad-spectrum antibiotic, antifungal and also as a moderate antitumor agent (Cheng et al., 2010; Yuan et al., 2013). To our knowledge, this is the first report of using *S*. *violaceusniger* AC12AB producing azalomycin as biological control agent in an agriculture system.

For a successful biocontrol agent, it is important to acquire root colonization ability so that the secondary metabolites produced by microorganisms would be available to the plant roots system (Johnston-Monje and Raizada, 2011; Larkin et al., 2011). Plant growth promoting *Streptomyces* (PGPS) are important microorganisms to develop a successful beneficial interaction between plants and microbes in a rhizoplane. PGPS are preferred over other plant growth promoting bacteria due to their enhanced colonization ability, their effect as biofertilization, biostimulation, and bioprotection (Saharan and Nehra, 2011; Rajput et al., 2013; Jog et al., 2014; Qiao et al., 2014). In current study, we demonstrated that *S*. *violaceusniger* AC12AB had the ability to produce plant growth hormone IAA along with its ability to produce siderophores and solubilize phosphates. Therefore, potato tubers treated with *S*. *violaceusniger* AC12AB developed in terms of root and shoot growth, tuber weight and better yield.

Results of greenhouse and field trials showed up to 90 and 80%, respectively, decrease in CS disease severity was observed when potato tubers inoculated with *S*. *violaceusniger* AC12AB. These results coupled with agar plate assay may explain the role of azalomycin as an antagonistic agent against PCS pathogens. Moreover, more than 25% increased yield was observed which could be attributed to the enhanced colonization ability of *Streptomyces*, production of plant growth hormones, siderophores, nitrogen fixation, and phosphate solubilization potential. Application of this type of bacteria will greatly enhance the production of potato and profit, which is especially important in developing countries.

## Author Contributions

AS conducted all experimental work. ZL assisted with project development and data analysis. SZ and AB assisted in performing NMR analysis. JH assisted in manuscript writing.

## Conflict of Interest Statement

The authors declare that the research was conducted in the absence of any commercial or financial relationships that could be construed as a potential conflict of interest.
