# Supplementary material for: A Potential Biocontrol Agent Streptomyces violaceusniger AC12AB for Managing Potato Common Scab
Source: Front Microbiol. 2019 Feb 8;10:202. doi: 10.3389/fmicb.2019.00202 (PMC6375851; doi:10.3389/fmicb.2019.00202)
Supplement: Supplementary file 1 [file Data_Sheet_1.docx]

**SUPPLEMENTARY FIGURES**


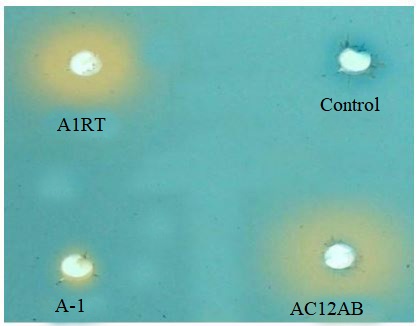


**Figure S1:** Siderophore productions showing orange colored zone around bacterial cultures.


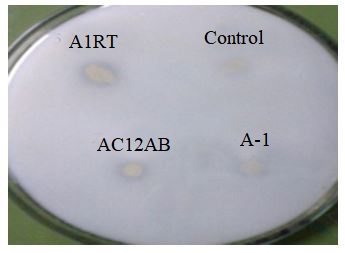


**Figure S2:** Phosphate solubilization seen as clear zone around bacterial cultures

**Figure S3:** HPLC-DAD-MS chromatogram showing molecular mass of the bioactive compound


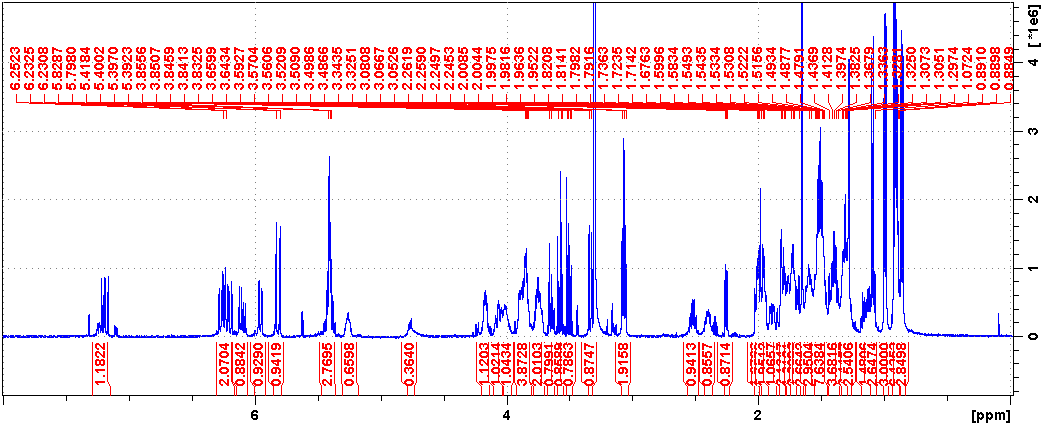


**Figure S4:** ^1^H-NMR spectrum of azalomycin (CD_3_OD, 400 MHz)


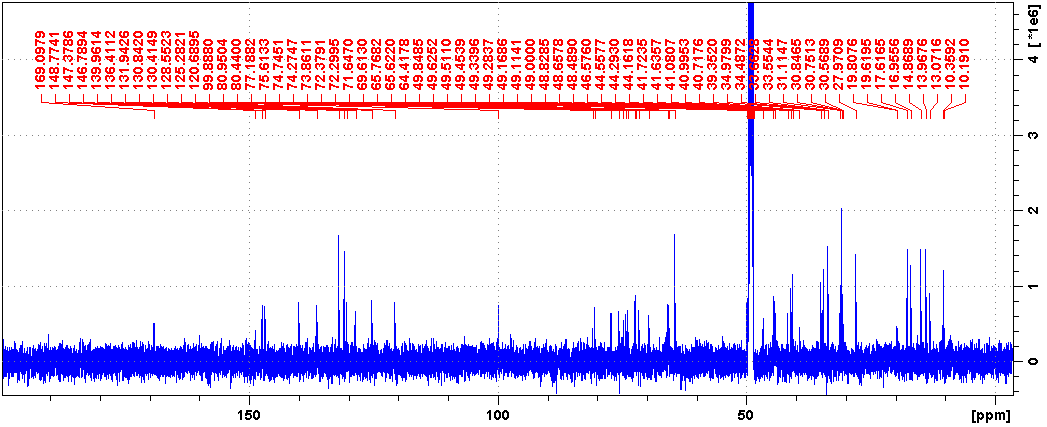


**Figure S5:** ^13^C-NMR spectrum of azalomycin (CD_3_OD, 100 MHz)


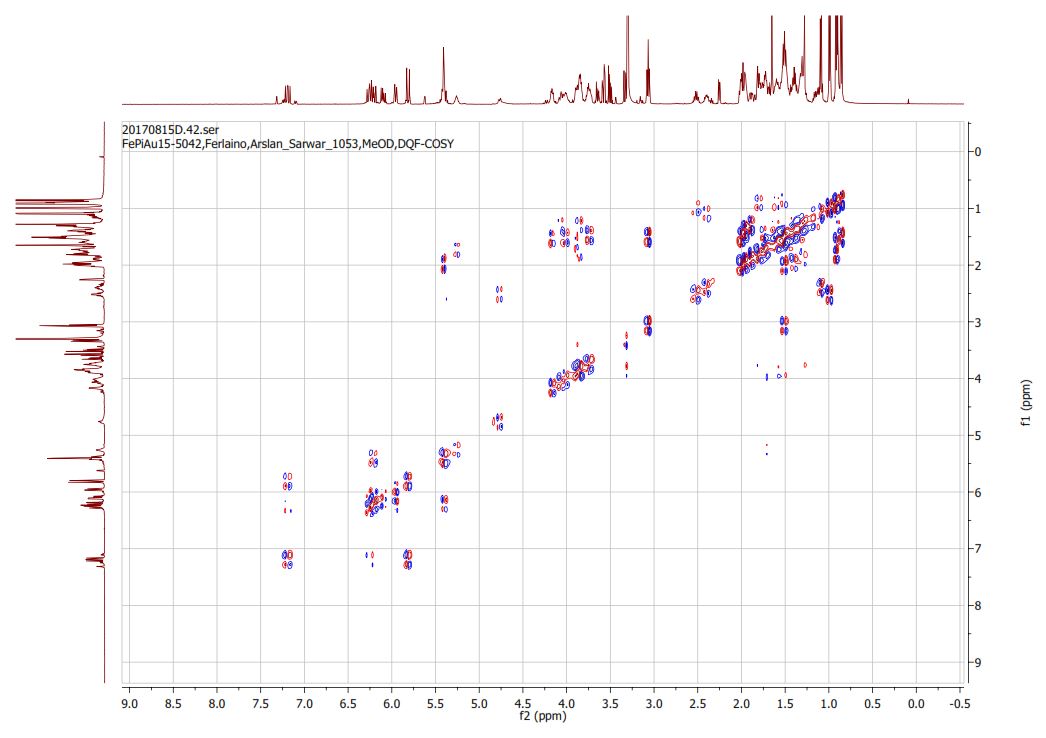


**Figure S6:** ^1^H/^1^H-COSY (Correlation Spectroscopy) spectrum of azalomycin.


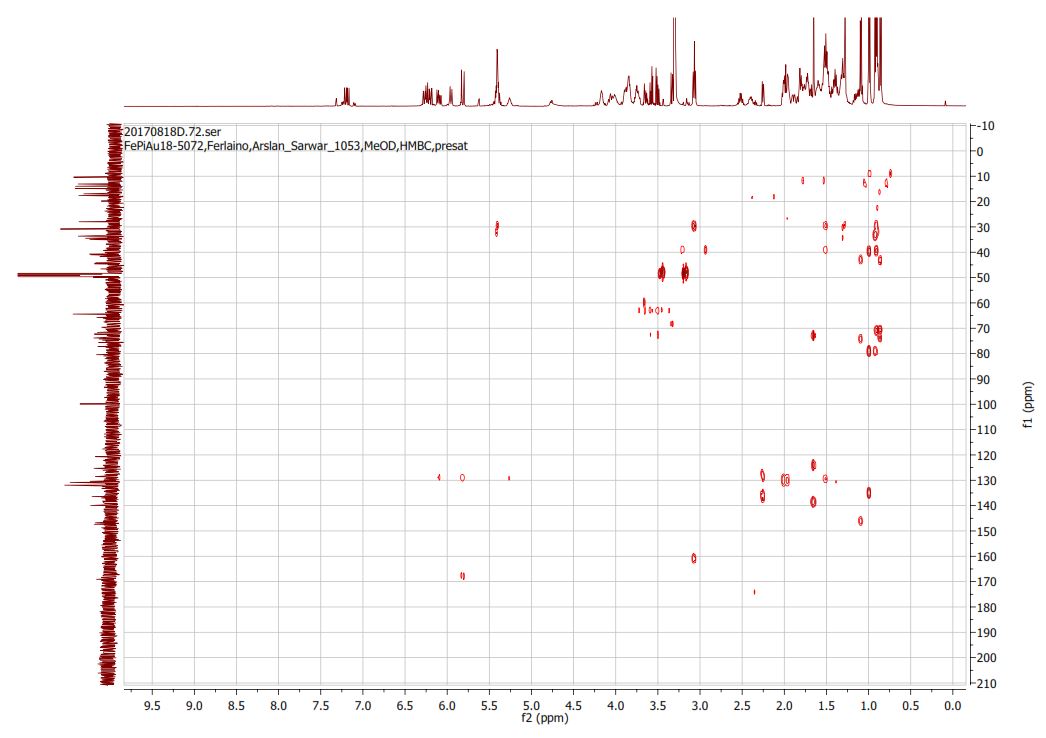


**Figure S7:** ^1^H-^13^C-HMBC spectrum of azalomycin.


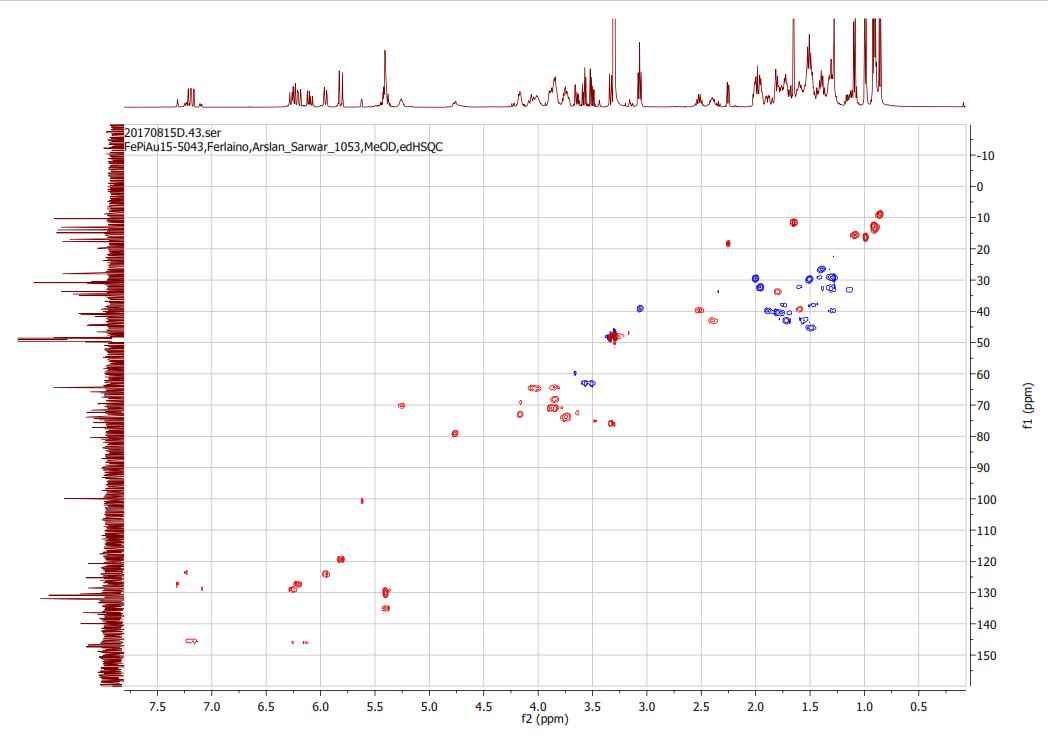


**Figure S8:** HSQC (Heteronuclear Single Quantum Correlation) spectrum of azalomycin.

**Figure S9:** UV-absorbance spectrum of bioactive compound

**SUPPLEMENTARY TABLES:**

**Table S1:** Antagonistic bacteria strains showing zone of inhibitions in mm

| Isolates | Name of the isolates | Zone of inhibition (mm) against *Streptomyces scabies* |
| --- | --- | --- |
| AC12AB | *Streptomyces* spp. | 18 |
| A1RT | *Streptomyces* spp. | 26 |
| A-2 | *Streptomyces* spp. | 12 |
| A-3 | *Streptomyces* spp. | 6 |
| A-4 | *Streptomyces* spp. | 6 |
| A-5 | *Streptomyces* spp. | 14 |
| 14C | *Streptomyces* spp. | 10 |

**Table S2:** antagonistic bacterial strains showing nitrogen fixation potential analyzed by acetylene reduction assay (ARA) with Gas Chromatography equipment.

| Sr. No. | Isolate Name | Ethylene produced (nMole/24h) |
| --- | --- | --- |
| 1 | *S. violaceusniger* AC12AB | 4351.0 |
| 2 | *Streptomyces* A1RT | 2278.0 |
| 3 | *Streptomyces* A-1 | 1549.0 |

**Table S3:** Tabular representation of NMR signals data

| **No** | **^1^H (δ, mult., *J*)** | **^13^C** | **^1^H-^13^C HMBC** |
| --- | --- | --- | --- |
| **1** |  | 169.1 |  |
| **2** | 5.8 (1H, d, J = 15 Hz) | 120.7 | 1 |
| **3** | 7.2 (1H, dd, J =10, 15 Hz) | 147 | 1 |
| **4** | 6.3 (1H, dd, J = 10, 15 Hz) | 130.4 |  |
| **5** | 6.2 (1H, dd, J = 8.7, 15 Hz) | 148 |  |
| **6** | 2.4 (1H, m) | 46.6 |  |
| **6-Me** | 1.1 (3H, d, J= 6.8 Hz) | 16.9 |  |
| **7** | 3.75 (1H, m) | 75.6 |  |
| **8** | 1.44, 1.74 (2H, m) | 39.4 |  |
| **9** | 3.79 (1H, m) | 74.7 |  |
| **10-Me** | 0.87 (3H, d, J= 6.8 Hz) | 10.4 |  |
| **11** | 3.89 (1H, m) | 72.3 |  |
| **12** | 1.34, 1.10 (1H, m) | 33.6 |  |
| **13** | 1.29 (2H, m) | 30.8 |  |
| **14** | 1.59 (1H, m) | 41.6 |  |
| **14-Me** | 0.91 (3H, d, J= 6.8Hz) | 14.8 |  |
| **15** | 3.86 (1H, m) | 72.2 |  |
| **16** | 1.83 (2H, m) | 41.7 |  |
| **17** |  | 99.9 |  |
| **18** | 3.34 (1H, d, J= 9.3 Hz) | 77.2 |  |
| **19** | 3.86 (1H, m) | 69.6 |  |
| **20** | 1.3, 1.9 (2H, m) | 41.1 |  |
| **21** | 4.08(1H, m) | 65.7 |  |
| **22** | 1.5, 1.6 (2H, m) | 44.6 |  |
| **23** | 5.23 (1H, m) | 71.6 |  |
| **24** | 1.65-1.7 (2H, m) | 44.1 |  |
| **25** | 3.86 (1H, m) | 65.6 |  |
| **26** | 1.58 (2H, m) | 44.2 |  |
| **27** | 3.86 (1H, m) | 66.3 |  |
| **28** | 1.58 (2H, m) | 40.7 |  |
| **29** | 4.16 (1H, m) | 74.2 |  |
| **30** |  | 139.9 |  |
| **30-Me** | 1.64 (3H, s) | 13.1 |  |
| **31** | 5.9 (1H, d, J= 10.7Hz） | 125.3 |  |
| **32** | 6.2 (1H, d, J= 10.7, 15.2Hz） | 128.5 |  |
| **33** | 5.4 (1H, d, J= 88, 15.2 Hz） | 136.4 |  |
| **34** | 2.5 (1H, m） | 41 |  |
| **34-Me** | 0.99 (3H, d, J= 6.9Hz) | 17.6 |  |
| **35** | 4.75(1H, dd, J= 3.9, 8.8Hz） | 80.4 |  |
| **36** | 1.77 (1H,m) | 35 |  |
| **36-Me** | 0.92 (3H, d, J= 6.8Hz) | 13.9 |  |
| **37** | 1.1 (2H, m) | 34.5 |  |
| **38** | 1.36 (2H, m) | 27.9 |  |
| **39** | 1.85-2.0 (2H, m) | 33.6 |  |
| **40** | 5.44 (1H, m) | 131.9 |  |
| **41** | 5.43 (1H, m) | 130.8 |  |
| **42** | 2.06 (2H,m ) | 30.7 |  |
| **43** | 1.62 (2H, m） | 30.5 |  |
| **44** | 3.08 (2H, t, J= 6.9Hz) | 41.9 | N-CN_2_ |
| **N-CN2** |  | 158.3 |  |
| **1'** |  | Weak signal |  |
| **2'** | 3.28 (2H, m) | 49.7 |  |
| **3'** |  | Weak signal |  |
